# Is it the Past or the Present? Employment Quality, Unemployment History, Psychological Distress and Mental Wellbeing in the United Kingdom

**DOI:** 10.1177/27551938241288788

**Published:** 2024-10-22

**Authors:** Rebeka Balogh, Deborah De Moortel, Sylvie Gadeyne, Julie Vanderleyden, Chris Warhurst, Christophe Vanroelen

**Affiliations:** 1 Brussels Institute for Social and Population Studies (BRISPO), Department of Sociology, 70493 Vrije Universiteit Brussel, Brussels, Belgium; 2 366511 Institute for Employment Research, University of Warwick, Coventry, UK; 3 54507 Flanders Research Foundation, Brussels, Belgium

**Keywords:** employment quality, precarious employment, unemployment history, mental health, United Kingdom, latent class analysis

## Abstract

Low employment quality and precarious employment have been associated with adverse mental health outcomes, yet the extent to which this association may be explained by the experience of unemployment “scarring” has not yet been explored. From a life course perspective, understanding this possible confounding is necessary. Drawing on the United Kingdom's Understanding Society dataset and using latent class analysis, we derived a typology of employment quality across six dimensions and assessed the links between individuals’ employment quality, unemployment history, and mental well-being and psychological distress. Our results show that precarious types of employment as well as a higher quality “protected part-time” were linked to low mental well-being, though important gender differences were noted. Accounting for past unemployment did not fully explain these associations. No such adverse associations were observed for increased psychological distress. Our results help further the understanding of employment quality as a social determinant of health and highlight the need for both life course and gender-sensitive research in this area.

Being employed is generally associated with better health and mortality outcomes than being unemployed.^1–3^ At the same time, there are considerable health inequalities among those in employment. Health inequalities exist with respect to the physical work environment,^4^ and evidence synthesized in a review of systematic reviews and meta-analyses underlines links between psychosocial risk factors for stress at the workplace and mental health and cardiovascular outcomes in particular.^5^ Recently, however, studies have pointed to the role of employment quality, and in particular precarious employment as a form of low-quality employment as a third work-related determinant of health that play a role alongside physical and psychosocial risk factors.^6–8^ Evidence on work-related health inequalities in general has been accumulating to the extent that questions are being asked about whether being employed in certain low-quality employment and work is better health-wise than having no job.^9,10^ However, evidence also suggests that it is not only current employment but also past unemployment that has implications for current mental health and well-being; that is, past exposure to unemployment potentially leaves “scars” on later mental health and well-being.^11,12^ Moreover, life course theory suggests that experiences and risk factors (including those related to employment) can accumulate over individuals’ life course.^13,14^ As such, the mental health associations of one's current job might also (partly) reflect that of previous employment experiences. This potential issue of past confounding has been overlooked in the cross-sectional research on employment quality so far. The aim of our investigation is therefore twofold: first, we explore mental health and well-being-related inequalities focused on employment quality using U.K. survey data (Understanding Society) and, second, we assess whether accounting for past labor market experience in the form of unemployment histories can explain the cross-sectional association between employment quality and mental health and well-being at the time of survey. As such, our study aims to help contextualize prior research which has shown a mental health disadvantage for those in precarious and low-quality employment^6,7^ by providing a life-course perspective.

## Employment Quality and Mental Health

Job quality encompasses both work quality (associated more closely with the tasks of the job) and employment quality (encompassing employment conditions, such as pay, security, aspects relating to working time, as well as employment relations).^15,16^ Here, we focus on employment quality, which, as an overarching and multidimensional concept,^17^ consolidates previously fragmented approaches that examined separate aspects of employment, such as temporary work, long working hours, and nonstandard working time in relation to health outcomes.^16^ With that, employment quality captures the multitude of ways in which labor market flexibilization across industrialized economies has impacted employment arrangements, with deviations from the traditional stable and secure, full-time standard employment relationship (SER) more prominent in the postwar period.^16,18,19^ While the SER represents an ideal type of high employment quality, precarious forms of employment are typically regarded as at the “lower end” of employment quality.^7,8^ As such, precarious employment goes beyond the presence of a temporary contract as it represents an accumulation of multiple aspects of low employment quality, such as insecurity, low pay, and lack of rights.^20^ Multidimensional exercises measuring and separating precarious forms of employment from employment arrangements more closely resembling the SER in contemporary labor markets are possible through typological, clustering methods.^18^ These typological methods, however, also allow for other ideal types of employment arrangements, which consist of a combination of favorable and unfavorable characteristics, to be identified as distinct from both the SER and precarious employment.^18^

In contrast to the physical and psychosocial aspects of work tasks, factors related to employment quality and precarious employment are often overlooked health determinants.^16,21^ Nonetheless, empirical evidence is emerging showing the importance of employment quality for employees’ mental health and well-being. A cross-national investigation of E.U. countries uncovered a typology of employment quality and highlighted mental health inequalities across the typology, with those in employment classified as “precarious” reported to be particularly affected.^6^ Similar country-specific typological studies have also since been carried out.^10,22–25^ Inequalities along employment quality types or trajectories have been observed in these studies for frequent mental distress using US survey data,^25^ for poor mental health in Belgian survey data,^10^ for moderate mental illness using US survey data,^23^ and using Belgian^22^ and Swedish^24^ register data looking at mental health-related disabilities, and common mental disorders, substance use disorders and suicide attempts, respectively.

This paper focuses specifically on mental health and well-being outcomes, as ill mental health represents a leading contributor to the global ill-health burden.^26^ It has been pointed out, however, that the notion of positive mental health or mental well-being remains important from a population health perspective and should be considered alongside mental ill-health^27^; “mental wellbeing and mental illness may represent two different but correlated continua,”,^28^ p. 461) in other words, it has been argued that a lack of mental ill-health might not necessarily represent (high) positive mental well-being. Though it has been suggested that scales measuring mental well-being and psychological distress (possible mental ill-health) in effect measure the same construct, a variety of items and scales are advised to be considered,^29,30^ particularly as a differential social gradient can also exist.^28^

## Political Economy of Health, the Life Course Perspective, and Embodiment

Understanding the role employment quality plays in shaping health inequalities can complement empirical evidence on class and socio-economic differences in health. Low employment quality and precarious employment can be thought of as “unequally distributed risk factors” that contribute to the reproduction of health inequalities between groups.^21,31^ Moving beyond such individual risk factors, however, (precarious) employment relations also form part of a broader political economy of health and of societal health determinants,^32^ and can be seen as a manifestation of unequal power relations which contribute to mental health inequalities.^33^

Analyses that incorporate past labor market experience when looking at present employment quality are, however, needed to further understand the role of (low) employment quality as a health determinant. Such analyses can, importantly, consider that individuals’ health may have been already shaped by previously “embodied” experiences.^34,35^ As a result, the previously established cross-sectional link between low-quality employment and adverse mental health and well-being may in fact be (partly) attributable to past (employment) experiences across the life-course which need to be disentangled.^13,14^ Longitudinal analyses can help in doing so.^36^ The few trajectory-based investigations published on the association between employment quality and mental health have indeed shown that, similarly to cross-sectional typologies of employment quality, typologies of multidimensional employment trajectories are also possible to distil, subsequently relating trajectories of (precarious) employment (and including unemployment) to workers’ health and well-being.^22–24^ Studies making use of longitudinal data have also started delving into matters of causality; indeed, not only can low employment quality and precarious employment lead to ill-health, but the reverse may also be true,^21^ though evidence causally linking precarious employment to ill-health is emerging. A recent study drawing on Swedish register data and considering multiple indicators of employment quality has shown that a switch from precarious to higher-quality employment has a causal effect on a reduction in all-cause mortality.^37^

Investigations to date, however, have rarely considered yet ought to account for the interrelation between (past) unemployment and (present) employment arrangements. Unemployment is an important psychosocial stressor and can affect mental health through a potential loss or reduction in income.^1^ It has also been suggested that past unemployment leaves “scarring effects” on later well-being and mental health outcomes,^11,12^ see also.^38^ Additionally, past unemployment can influence present employment quality, potentially leading to employment in a temporary job^39^ or a future wage penalty.^40^

## The Case Study of the United Kingdom

There have been several concerns expressed not only about persistent and even widening health inequalities in the country, but also regarding the proliferation of low-quality/precarious employment and their implications for health and its social gradient in the United Kingdom.^2,41,42^ As a liberal market economy,^43^ the United Kingdom's labor market is characterized by high levels of flexibility, and issues of low pay, underemployment, and an overarching “one-sided flexibility” have been highlighted as key concerns for precarious employment.^42,44,45^ Examples of such “one-sided flexibility” include zero-hours and minimum-hours contracts, which do not guarantee any (or many) hours of paid work a week.^44^ In addition, there is a concern that the less generous provisions of the country's welfare state may not adequately cushion the health impacts of low employment quality.^46^ A recent study nevertheless did not find convincing evidence that workers in precarious employment in Anglo-Saxon welfare states may be more exposed to poor mental health than did workers in states with higher levels of decommodification, though sample size may have been a problem in this analysis.^47,48^ In sum, given concerns about labor market flexibility, widening health inequalities, as well as evidence highlighting the particularly high prevalence of a type of precarious employment characterized by a multitude of adverse characteristics (including low pay and involuntary part-time work),^18^ investigations focusing on employment quality and health in the United Kingdom are warranted. Yet, to the best of our knowledge, no effort has been made to operationalize a typology of employment quality indicators specifically for U.K. data to uncover the distribution and configurations of employment quality and its health implications of, precarious employment, as has been done for other countries.^10,22–25^

### The Aim of this Article

In light of the identified research gap and the need to study this problem in the U.K. context, this article investigates the following two research questions: (*a*) How does employment quality relate to mental health outcomes in the United Kingdom? And (*b*), what happens to these associations when accounting for prior unemployment?

## Methods and Data

### Data

Data for our analyses is derived from the Understanding Society survey, a longitudinal study in the United Kingdom that follows a panel of respondents over time and interviews them and any household members on various aspects of individual and household life.^49^ Our main analysis focuses on the fourth wave, which was conducted between January 2012 and June 2014, while information from the preceding three waves and wave five is also used. In wave four, the rotating working conditions module was administered, which allowed us to gather information on a broad range of employment quality indicators, and choosing wave four as opposed to wave two meant we were able to minimize the “backdating” of lifetime unemployment information (given the fact that the employment status history module was administered to some in wave five), and to balance sample size (considering attrition). It was also over 2012–2014 that both part-time working and working hours underemployment peaked in the United Kingdom following the global financial crisis-triggered recession, and zero-hours contracts started sharply increasing (which partly may be due to increased reporting),^2,42^ with clear relevance for employment quality and related well-being, making it an insightful time point for our analyses. The University of Essex Ethics Committee approved Understanding Society's main survey data collections.

### Study Sample

Individuals aged 25 to 60 years at the time of the wave four interview who had worked as employees in the preceding week or otherwise had a paid job as an employee were eligible to be included in the analyses. While employment quality has previously been operationalized to include self-employed groups in other countries,^50^ our analysis focuses on employees only due to their distinct contractual relationship and related employment characteristics. Individuals on maternity leave and those working in the armed forces^47^ were excluded, as were a small number of observations (around 2% of the sample) whose main activity was not “in employment” (e.g., students). The number of valid observations (with a nonzero self-completion weight) used for the Latent Class analyses was 7,507 (where the wave four longitudinal weight was used) while the sample comprises 6,269 valid observations for the subsequent regression analyses (with the wave five longitudinal wave applied and excluding observations with incomplete covariate information). A flow chart of the sample selection is shown in Figure S1.

### Employment Quality

We operationalized 12 employment quality indicators in the Understanding Society sample related to the following six dimensions: employment security (contract type and multiple job-holding), income (pay level, non-wage benefit, and pay progression), workers’ rights (uncompensated overtime), working time (working hours, irregular/nonstandard working time, and informal working time flexibility), employability (any recent training) and collective representation (see Table 1). This approach was inspired by earlier empirical studies using a similar approach^6,10,18,25,51–54^ as well as corresponding literature reviews.^16,20^ Efforts were made to consider the specific U.K. context while selecting and constructing the indicators (e.g., potential underemployment indicator reflected the “living hours” concept with a minimum of 16 h a week).^55^ Characteristics pertain to the individuals’ main job, except for labor income (henceforth “pay”) and long working hours which represent main and side jobs if present, and we also included an additional indicator on multiple jobholding.

**Table 1.** Overview of employment quality indicators.

Dimension	Indicator	Question(s)/variable(s) used for indicator	Categories
**1. Employment security**	1.1 Contract type	Leaving aside your own personal intentions and circumstances, is your job permanent or is there some way it is not permanent?	a. Permanent b. Temporary
	1.2 Multiple jobholding	Do you currently earn any money from a second job, odd jobs, or from work that you might do from time to time, apart from any main job you have?	Single jobholder Multiple jobholder
**2. Income**	2.1 Relative pay level	Quartiles of monthly labor income, adjusted for inflation (as wave spans over 2 years)	Lowest quartile 2nd/3rd quartile Highest quartile
	2.3 Nonwage benefit	Does your employer offer a pension scheme (…) AND Do you belong to your employer's pension scheme?	a. Yes b. No/don’t know
	2.4 Pay progression	Some people can normally expect their pay to rise every year (…) Are you paid on this incremental scale?	a. Yes b. No/don’t know
**3. Workers’ rights**	3.1 Uncompensated extra time	How many hours overtime do you usually work in a normal week? AND How much of that overtime is usually paid overtime?	No unpaid overtime Any unpaid overtime
**4. Working time**	4.1 Long working hours (overall)	Thinking about your main job, how many hours excl. overtime & meal breaks are you expected to work in a week? AND hours in 2nd job AND overtime	48 or less more than 48
	4.2 Potential underemployment (in main job)	Thinking about your main job, how many hours excl. overtime & meal breaks are you expected you work in a week?	16 or more less than 16
	4.3 WT irregularity/nonstandard WT	Which time of day do you usually work? AND Do you ever work at weekends?	No irregularity, standard WT Rotating shifts or WT varies/no usual pattern/night work/regular weekend work
	4.4 Informal WT flexibility	Are you able to vary your working hours on an informal basis, e.g., by rearranging your start or finish times if you need to?	a. Yes b. No
**5.Employability**	5.1 Training	Has done some training since last interview, and this was provided by the employer either on or off the job (listed among 3 longest trainings if n of trainings > 3)	Recent employer-provided training No recent employer-provided training
**6. Collective organization**	6.1 Collective representation at the workplace	Is there a trade union, or a similar body such as a staff association, recognized by your management for negotiating pay or conditions for the people doing your sort of job in your workplace?	a. Yes b. No

Note: WT = working time.

### Mental Health Outcomes

Our investigation focused on two mental health-related outcomes: psychological distress and low mental well-being.

Psychological distress is assessed using the 12-item General Health Questionnaire (GHQ), containing six negatively and six positively worded statements.^56^ We applied the GHQ scoring method, which results in a score ranging from 0 to 12.^56^ A cut-off value of 3 or more was chosen as indicating possible psychological distress in line with previous studies and given the score's distribution in our analytical sample,^57,58^ while sensitivity analyses with a score of 4 or more representing psychological distress were also conducted.^59^

Low mental well-being is represented by the Short Warwick-Edinburgh Mental Well-being Scale (SWEMWBS).^60^ The original scale was developed (and validated) to capture the concept of “positive mental health” (also called “mental well-being”).^27^ The scale contains seven positively worded items and respondents can choose whether each of the seven items have applied to them in the preceding two weeks: none of the time, rarely, some of the time, often, or all of the time.^60^ Higher summed scores indicate higher levels of well-being and a score of 19.25 or below on the transformed metric scale was chosen to be indicative of low mental well-being,^61^ with additional sensitivity analyses conducted with an alternative cutoff point.

### Unemployment History

Deriving the length of past unemployment involved a number of steps and was partially guided by the work of Wright.^62^ A Lifetime Employment Status History module eliciting lifetime spells of activities since individuals left full-time education was incorporated in the Understanding Society survey, with the module administered to some households in the first wave and to some in the fifth wave. For respondents who completed the module in the first wave, assessment of their lifetime unemployment was then complemented by assessing any potential unemployment spells between the first and fourth waves with the help of the Annual Event History modules, which elicited activity spells in between waves of questionnaires. For respondents who completed the Lifetime Employment Status History module in wave five, their activity spells were “backdated” to the date of their wave four interview. A binary variable of having been unemployed in the past and a continuous variable representing the length of past unemployment (in months) to assess a dose-response relationship were included in the analyses.^63,64^

### Analytical Approach

To derive ideal types of employment quality arrangements across our sample, latent class analysis (LCA) was applied. LCA identifies latent groups (clusters) that share similar features based on manifest indicators—in our case, employment quality.^65^ These groups are described by item response probabilities that characterize clusters, in this case, probabilities that employees within that group hold attributes such as a temporary contract or collective representation at their workplace.^65^ The technique has been used previously to create typologies of employment quality and to identify workers in precarious employment.^6,10,18,24,25,50,51^ Models with two to nine clusters were run. To determine the ideal number of clusters, we employed various methods such as the Akaike Information Criterion, the Bayesian Information Criterion, the Vuong-Lo-Mendell-Rubin likelihood ratio test, and measures of entropy along with theoretical considerations and the results of prior typologies.^65,66^ After examining the bivariate residuals, residual associations were also tested and included in the best fitting latent class models between the two working hours indicators and unpaid overtime and pay, respectively (these characteristics are strongly linked).^67^

Subsequently, a modal assignment was used wherein individuals were assigned to the employment quality cluster to which they have the highest probability of belonging.^65^ A joint typology was created for men and women to allow for comparisons to be made between the emerging employment quality types, and regression analyses were then run separately given gender differences in mental health and employment.^68^ The resulting categorical variable representing the employment quality typology and variables related to unemployment history were then included in logistic regression models as predictors. We assessed associations between the employment quality typology and the two mental health outcomes (Model 1), then between unemployment history and the mental health indicators, both as a dichotomous indicator (Model 2), and to test a dose response relationship (Model 3). Model 4 included the employment quality typology and adjusted for covariates, whereas Model 5 additionally included the dichotomous variable representing whether an individual had a prior unemployment history (full model). All models adjusted for age, and Models 4 and 5 included partnership status (whether the person was married/living as a couple or not), highest educational attainment (degree level/equivalent, A-level/equivalent, General Certificate of Secondary Education (GSCE)/equivalent, other/no qualification), and ethnic background (white British, white non-British, mixed/other, Asian/Asian British, black/black British)^59^ as covariates. We also modeled the length of past unemployment as a percentage of careers as an additional robustness check. The goodness of fit of the regression models was assessed using a graphical approach^69^ and the Hosmer-Lemeshow test.^64^ LCA was conducted in Mplus^66^ while data cleaning and all other analyses were performed using R.^70^ Code for analyses is available on https://github.com/rebekabalogh/past_and_present.

## Results

Descriptive statistics of the LCA subsample are shown in the Supplementary Material (Table S1). Men and women were evenly represented, and the mean age of employees was 42.

### Employment Quality Typology

Model fit indices for the LCA are presented in Table S2. We weighed up both the models’ fit and parsimony as shown by model fit indices^65^ and their alignment (or lack thereof) with theory and cross-national results of employment quality typologies. After a careful inspection of different cluster solutions, particularly those highlighted to be potentially best-fitting by the information criteria and the Vuong-Lo-Mendell-Rubin likelihood ratio test, the six-cluster solution with residual associations was deemed to be optimal as it fared best both in terms of model fit and parsimony while best highlighting and separating higher quality as well as precarious arrangements. The quality of classification in terms of modal assignment (the average latent class probabilities for each cluster) for the final cluster solution is presented in Table S3. Item response probabilities describing the employment quality characteristics of each of the six clusters, along with the distribution of the employment characteristics in the overall sample, are shown in Table S4. We labelled the clusters to highlight some of their distinct characteristics. Given similarities with prior employment quality typologies, some of the cluster labels were retained from these earlier studies.^6,10,18,24,25^

The first group is characterized by a relatively high probability of unfavorable employment quality in terms of pay, temporary contract, nonwage benefit, training, collective representation at the workplace, as well as potential underemployment and pay progression. To emphasize the multitude of unfavorable conditions associated with this cluster, particularly low wages and potential underemployment, the label “Precarious Unsustainable” was used for this group.^18^

The second group is characterized by several favorable conditions regarding pay, permanent contract, nonwage benefit, collective representation, and training. What sets this group apart, however, is a simultaneous high probability of working irregular or nonstandard hours and the highest relative probability of long working hours among all groups. This group is also most likely to be multiple jobholders out of all the clusters and not be able to arrange their working hours on an informal basis. Cross-tabulations with broader work quality indicators (see Table S5) show that employees in this group also have lower perceived autonomy over their working hours. To emphasize the effort of nonstandard and long working hours and inflexibility, this group is labelled “High Effort”.^71^

The third cluster resembles the Precarious Unsustainable group with regards to pay and working time (as shown in Table S5, the average number of weekly working hours in their main job is 28), yet it is characterized by more advantageous features such as nonwage benefits and collective representation, hence it received the label “Protected Part-time.”

Lower wage yet higher than average probabilities of long and irregular/nonstandard hours as well as unfavorable features in terms of nonwage benefit and pay progression characterize the fourth cluster. To highlight these important distinguishing features of this group that set it apart from others, but particularly the SER, it is labelled “Precarious Intensive”.^18^

The fifth cluster bears resemblance to the High Effort group. However, employees in this group tend to have a much higher probability of being able to adjust their working hours for personal reasons, and they have regular and standard working times. At the same time, this cluster is also characterized by near-complete lack of collective representation at their main workplace and higher probability of undertaking unpaid overtime than employees in other groups. To highlight similarities with the cross-national Portfolio cluster,^18^ we have retained this label for this group.

The last group consists of employees in mostly favorable arrangements in terms of employment stability, wage, collective representation, and working time, although we note that the probability of uncompensated overtime is still considerable in this group. Given that this group most resembles what has been termed the “Standard Employment Relationship” (SER), we have used this label for this group.^18^

Overall, important differences can be observed for the gender composition of different clusters, with the SER (53%) and Precarious Unsustainable (60%) cluster being slightly, and the Protected part-time (73%) cluster being most, female-dominated (Table S5). Notable differences also emerged when looking at major groups of occupations.

### Mental Health Associations

Subsequent regression analyses (presented in Tables 2 and 3) show that men in a Portfolio-like job had lower odds of experiencing psychological distress compared to men in an SER-like arrangement, even after accounting for past unemployment and other covariates. None of the other groups, however, had significantly higher (or lower) odds of psychological distress among men, and there was no significant association between past unemployment and present psychological distress. Among women, none of the employment quality clusters showed associations with psychological distress compared to the SER. Past unemployment experience, however, was linked to higher odds of experiencing current psychological distress among women (not in a dose response manner), although this association was no longer present in the fully adjusted model. Inequalities in mental well-being along the lines of employment quality were more pronounced than those in psychological distress. Men in the Precarious Unsustainable, Protected Part-time and the High Effort groups had higher odds of reporting low mental well-being than their counterparts in an SER-like job. This association was attenuated after adjustment but remained statistically significant for the Precarious Unsustainable and Protected Part-time clusters but was no longer statistically significant for the High Effort group. The Portfolio group among men, on the other hand, showed lower odds of low mental well-being after adjustment. Among women, the two Precarious clusters and the Protected Part-time group were associated with lower mental well-being. Independently of employment quality, among both genders, past unemployment was also a predictor of lower mental well-being, although we did not find evidence for a dose response relationship. We also tested for interactions between past unemployment and the employment quality types but found no evidence for such interactions (results not shown).

**Table 2.** Associations between employment quality types, past unemployment, and psychological distress and mental well-being among men. Odds ratios (and 95% confidence intervals) from logistic regressions.

	**Psychological distress**					**Low-mental well-being**				
	*Model 1*	*Model 2*	*Model 3*	*Model 4*	*Model 5*	*Model 1*	*Model 2*	*Model 3*	*Model 4*	*Model 5*
**Employment quality type (Ref: Standard Employment Relationship)**	**-**	**-**	**-**	**-**	**-**	**-**	**-**	**-**	**-**	**-**
Precarious Unsustainable	1.17 (0.81, 1.70)			1.26 (0.85, 1.85)	1.25 (0.84, 1.84)	1.83*** (1.29, 2.60)			1.56* (1.08, 2.26)	1.54* (1.06, 2.24)
High Effort	1.14 (0.78, 1.66)			1.18 (0.81, 1.73)	1.19 (0.82, 1.74)	1.44* (1.01, 2.05)			1.32 (0.93, 1.88)	1.33 (0.94, 1.89)
Protected Part-time	0.99 (0.63, 1.58)			1.06 (0.65, 1.72)	1.05 (0.65, 1.71)	1.98** (1.29, 3.04)			1.71* (1.08, 2.68)	1.69* (1.08, 2.65)
Precarious Intensive	0.87 (0.61, 1.25)			0.94 (0.64, 1.37)	0.92 (0.63, 1.36)	1.31 (0.92, 1.84)			1.13 (0.78, 1.64)	1.10 (0.76, 1.60)
Portfolio	0.68* (0.47, 0.98)			0.65* (0.45, 0.94)	0.65* (0.45, 0.94)	0.68* (0.48, 0.97)			0.67* (0.47, 0.96)	0.67* (0.47, 0.96)
**Been unemployed in the past (Ref: No)**	**-**	**-**	**-**	**-**	**-**	**-**	**-**	**-**	**-**	**-**
Yes		1.17 (0.90, 1.51)	1.15 (0.86, 1.54)		1.16 (0.89, 1.50)		1.36** (1.08, 1.72)	1.30* (1.01, 1.66)		1.28* (1.01, 1.62)
**Past unemployment (in months)**			1.00 (0.99, 1.01)					1.00 (1.00, 1.01)		
Observations	2565	2565	2565	2565	2565	2565	2565	2565	2565	2565

Odds ratios (and 95% confidence intervals).

All models are adjusted by age, and Models 4 and 5 are additionally adjusted by partnership status, ethnic background, and educational attainment

*p < 0.05, **p < 0.01, ***p < 0.001

**Table 3.** Associations between employment quality types, past unemployment, and psychological distress and mental well-being among women. Odds ratios (and 95% confidence intervals) from logistic regressions.

	**Psychological distress**					**Low mental well-being**				
	*Model 1*	*Model 2*	*Model 3*	*Model 4*	*Model 5*	*Model 1*	*Model 2*	*Model 3*	*Model 4*	*Model 5*
**Employment quality type (Ref: Standard Employment Relationship)**	**-**	**-**	**-**	**-**	**-**	**-**	**-**	**-**	**-**	**-**
Precarious Unsustainable	0.79 (0.61, 1.03)			0.85 (0.64, 1.13)	0.84 (0.64, 1.12)	1.59*** (1.23, 2.04)			1.39* (1.05, 1.83)	1.37* (1.04, 1.81)
High Effort	1.03 (0.74, 1.43)			1.05 (0.76, 1.46)	1.06 (0.77, 1.47)	1.14 (0.81, 1.60)			1.12 (0.79, 1.59)	1.13 (0.80, 1.61)
Protected Part-time	0.93 (0.73, 1.20)			0.99 (0.76, 1.29)	0.99 (0.76, 1.29)	1.63*** (1.27, 2.09)			1.44* (1.09, 1.89)	1.43* (1.09, 1.89)
Precarious Intensive	1.08 (0.80, 1.45)			1.14 (0.83, 1.55)	1.13 (0.83, 1.54)	1.69*** (1.27, 2.25)			1.45* (1.09, 1.95)	1.44* (1.08, 1.93)
Portfolio	0.98 (0.73, 1.33)			1.00 (0.74, 1.35)	0.99 (0.74, 1.34)	1.06 (0.76, 1.48)			1.01 (0.72, 1.42)	1.01 (0.72, 1.41)
**Been unemployed in the past (Ref: No)**	**-**	**-**	**-**	**-**	**-**	**-**	**-**	**-**	**-**	**-**
Yes		1.27* (1.01, 1.59)	1.31* (1.02, 1.69)		1.24 (0.98, 1.56)		1.32* (1.05, 1.65)	1.26 (0.99, 1.60)		1.29* (1.03, 1.62)
**Past unemployment (in months)**			1.00 (0.99, 1.00)					1.00 (1.00, 1.01)		
Observations	3704	3704	3704	3704	3704	3704	3704	3704	3704	3704

Odds ratios (and 95% confidence intervals).

All models are adjusted by age, and Models 4 and 5 are additionally adjusted by partnership status, ethnic background, and educational attainment.

*p < 0.05, **p < 0.01, ***p < 0.001.

### Sensitivity Analyses

Sensitivity analyses show that using a higher cutoff to dichotomize the General Health Questionnaire scores for psychological distress does not affect the adjusted regression estimates meaningfully (see Tables S7 and S8). Lowering the threshold to below 19.25 on the SWEMWBS, which approximately captures the lowest 15% of the subsample^61^ results in more pronounced mental well-being inequalities for the employment quality typology; however, past unemployment is no longer significantly associated with low mental well-being (see Tables S9 and S10). We find that estimates for the employment quality typology are largely unaffected when taking over 6 or 12 months of total past unemployment as a threshold (instead of over 0 months), though only among women, not among men, does over 12 months of past unemployment predict low mental well-being independently of present employment quality (see Tables S11 and S12). Modelling the length of past unemployment as a percentage of working life did not affect the estimates of the employment quality clusters. However, a one percentage point increase in career spent in unemployment was only significantly associated with low mental well-being among women, and only so prior to further adjustment of present employment quality and covariates (see Tables S13 and S14).

## Discussion

We assessed present multidimensional employment quality and past unemployment history simultaneously in relation to employees’ mental health outcomes in the United Kingdom. To our knowledge, we are first researchers to present such a multidimensional employment quality typology specifically applied to U.K. data and to examine this association simultaneously in the context of unemployment histories’ scarring. Our analysis highlighted two precarious groups who were characterized by several unfavorable employment conditions, which together accounted for a significant proportion—over one third—of our sample. Overall, our results pointed to important gender-specific inequalities across the employee population in terms of low mental well-being. Women and men were often unequally represented in the different employment quality arrangements, highlighting the need to consider the gender dimension of employment quality and related mental health outcomes.^68^ Our findings need to be interpreted within a wider framework in which employment relations interact with, and are embedded in, other social and political determinants of health,^32^ and our findings also resonate with research showing how the unequal societal distribution of power can shape health inequalities.^33^

A mental well-being disadvantage was observed for the two precarious as well as the Protected Part-time groups. As such, our findings add to the growing evidence base on employment arrangements’ implications for mental health.^6,10,22–25^ Specifically, these inequalities observed point to the potential implications of (low) wages and underemployment,^2,42,45^ and long and nonstandard hours (and associated employment quality characteristics) for workers’ well-being in the United Kingdom.

From a policy perspective, the research findings underline the need to address aspects of “one-sided flexibility” in the United Kingdom, including and not limited to, by guaranteeing adequate number of working hours (“Living Hours”) and predictability to workers, addressing issues of low pay through a variety of measures available,^44,45,55^ as well as affording “Quality Part-time Work” to those on less than full-time hours.^72^

We also found that past unemployment generally does not (entirely) explain the present association between (low) employment quality and low mental well-being. Indeed, the odds of low mental well-being only marginally attenuated once we accounted for unemployment histories, though this may also be due to decreased unobserved heterogeneity resulting from the inclusion of a further predictor in the model.^73^ This, however, does not mean that individuals’ past (employment) scarring^11,12,38^ should be overlooked from a well-being perspective. Having accounted for both the past (unemployment) and the present (employment quality), our results, however, imply there may be a need to consider both, as the two at times were shown to have associations with mental well-being independent of the other (and with no evidence for an interaction between the two), though we note that this was not universally the case in all model specifications. We also need to point out that there is a possibility that an unemployment history (of length) reflects a precarious working history with past low-quality jobs,^22^ and there may also be sensitive or critical periods when having no (or a precarious) job may have adverse consequences,^13,14^ a possibility that future research might explore. Other “fixed” individual characteristics may also play a part.^74^ We also note that unemployment experiences can be greatly heterogeneous in terms of spell length or reasons for job loss,^75^ and their health impact is also dependent upon the wider economic context of a country.^76^ Nevertheless, while it is possible to “analytically separate” past and present, as well as employment from unemployment, a consolidated approach is needed that situates them in individuals’ working lives and beyond.^13^ Our study overall further reiterates the need for life course analysis to better understand the way individuals’ well-being is shaped by (employment-related) factors over time,^13,14,34,35^ and we hope to inspire future research that further disentangles temporal links between unemployment experiences, multidimensional employment quality, and mental health outcomes.

The lack of associations with regards to psychological distress for employment quality (apart from the Portfolio cluster, which was shown to be protective) and for unemployment histories (in the fully-adjusted models) may be partially explained by the so-called “healthy worker effect,” that is, that individuals that become and remain employed might have better (mental) health than the overall population^77^ and this effect may hold for mental ill-health more so than for low mental well-being, as the former could be more likely to lead to a so-called “health selection” out of employment than the latter. There may also be a selection effect in terms of resources among those who gain and keep employment due to the nature of the U.K. welfare state and more limited provisions such as social assistance or childcare services.^78^ However, our results, showing employment quality-related inequalities regarding mental well-being again suggest that both mental ill-health and mental well-being should be considered alongside employment quality,^28^ though we point out again that it has been suggested that the two scales we relied on in fact measure the same underlying construct.^29^ Direct comparisons between regression models with two different dependent variables (in this case, two mental health outcomes), however, are made difficult by differential unobserved heterogeneity.^73^ Our results on low mental well-being are nevertheless concordant with prior cross-national results on employment quality-related well-being across the European Union^6,50^; these studies relied on a well-being instrument highly correlated with the original Warwick-Edinburgh Mental Well-being scale.^27^

It is important to note the gender differences with respect to mental well-being in our analysis. Women who were most likely to belong to the Precarious Intensive cluster had significantly higher odds of reporting low mental well-being, unlike their male counterparts in the same group. It could be that the irregular or long hours coupled with the lower material reward of this cluster of employment quality particularly affects women as many might be balancing their paid work with (more) unpaid reproductive work responsibilities. These inequalities in unpaid labor might also help explain why the Portfolio group was shown to be “protective” in terms of both psychological distress and well-being outcomes among men, but not among women. Indeed, a recent systematic review suggested that unpaid work is associated with adverse mental health outcomes among women but less so among men,^79^ again emphasizing the need to account for work on all—not on paid alone—spectra.^68^ Both material deprivation and adverse psychosocial consequences are hypothesized pathways through which precarious employment is thought to affect mental health.^21^ There may be differences in the psychosocial implications these forms of employment may have on men and women, or in the way their earnings ultimately translate into household-level poverty.

Some limitations need to be stressed when interpreting our findings. First, we assigned individuals to the employment quality cluster to which, based on their posterior probabilities, they most likely belonged, and, as such, our regression models did not incorporate the measurement uncertainty inherent in LCA.^65^ Some overlap between classes, particularly between the two Precarious groups, can be observed (see Table S3), also suggested by the measure of entropy. Second, deriving lifetime unemployment from Understanding Society's modules is a complex undertaking and the corresponding indicators might reflect both measurement errors in terms of recall bias, and they are products of many assumptions that need to be made when using data from such complex modules.^62^ As a result, and as a third limitation, around 5 percent of our sample had missing values on past unemployment while our analysis was restricted to those who had complete information. The fit of adjusted regression model for low mental well-being among women could have been further improved. Finally, we focused on the wage-employed, and so we did not consider those that were presently unemployed or self-employed.

Our study, however, has considerable strengths as well. First, to our knowledge, we are the first to present a country-specific typology of employment quality using representative U.K. data. While trajectory-based studies drawing on administrative data can be hindered by a more restricted range of employment quality characteristics available for analysis,^22^ data from Understanding Society allowed us to consider a wide range of employment conditions and relations and to extend our longitudinal perspective by deriving unemployment histories. We also conducted gender-stratified analyses that pointed to differences regarding employment quality and well-being outcomes. In addition, we considered two mental health outcomes with differing underpinnings.

## Conclusion

The important contributions of this paper are twofold. First, the present investigation suggests that low mental well-being is unequally distributed across dimensions of employment quality among U.K. employees, particularly affecting the precarious, lower-paid, or part-time employees, and that this unequal distribution looks different for men and women. Second, this mental well-being disadvantage was not fully explained by past unemployment experience, though past unemployment in some cases may also be associated with lower present well-being. Our findings can shed light on employment quality and precarious employment as health determinants both in their own right and within individuals’ broader working lives.

## Supplemental Material

sj-docx-1-joh-10.1177_27551938241288788 - Supplemental material for Is it the Past or the Present? Employment Quality, Unemployment History, Psychological Distress and Mental Wellbeing in the United Kingdom Supplemental material, sj-docx-1-joh-10.1177_27551938241288788 for Is it the Past or the Present? Employment Quality, Unemployment History, Psychological Distress and Mental Wellbeing in the United Kingdom by Rebeka Balogh, Deborah De Moortel, Sylvie Gadeyne, Julie Vanderleyden, Chris Warhurst and Christophe Vanroelen in International Journal of Social Determinants of Health and Health Services
