# Supplementary material for: Is it the Past or the Present? Employment Quality, Unemployment History, Psychological Distress and Mental Wellbeing in the United Kingdom
Source: Int J Soc Determinants Health Health Serv. 2024 Oct 22;55(3):289–302. doi: 10.1177/27551938241288788 (PMC12171024; doi:10.1177/27551938241288788)
Supplement: sj-docx-1-joh-10.1177_27551938241288788 - Supplemental material for Is it the Past or the Present? Employment Quality, Unemployment History, Psychological Distress and Mental Wellbeing in the United Kingdom [file sj-docx-1-joh-10.1177_27551938241288788.docx]

Supplementary material

[Sub-sample selection 2](#_Toc171669313)

[Descriptive table for sub-sample 3](#_Toc171669314)

[Latent Class Analysis - model selection 4](#_Toc171669315)

[Characteristics of clusters 8](#_Toc171669316)

[Goodness of fit for regression models 11](#_Toc171669317)

[Sensitivity analyses 12](#_Toc171669318)

# Sub-sample selection


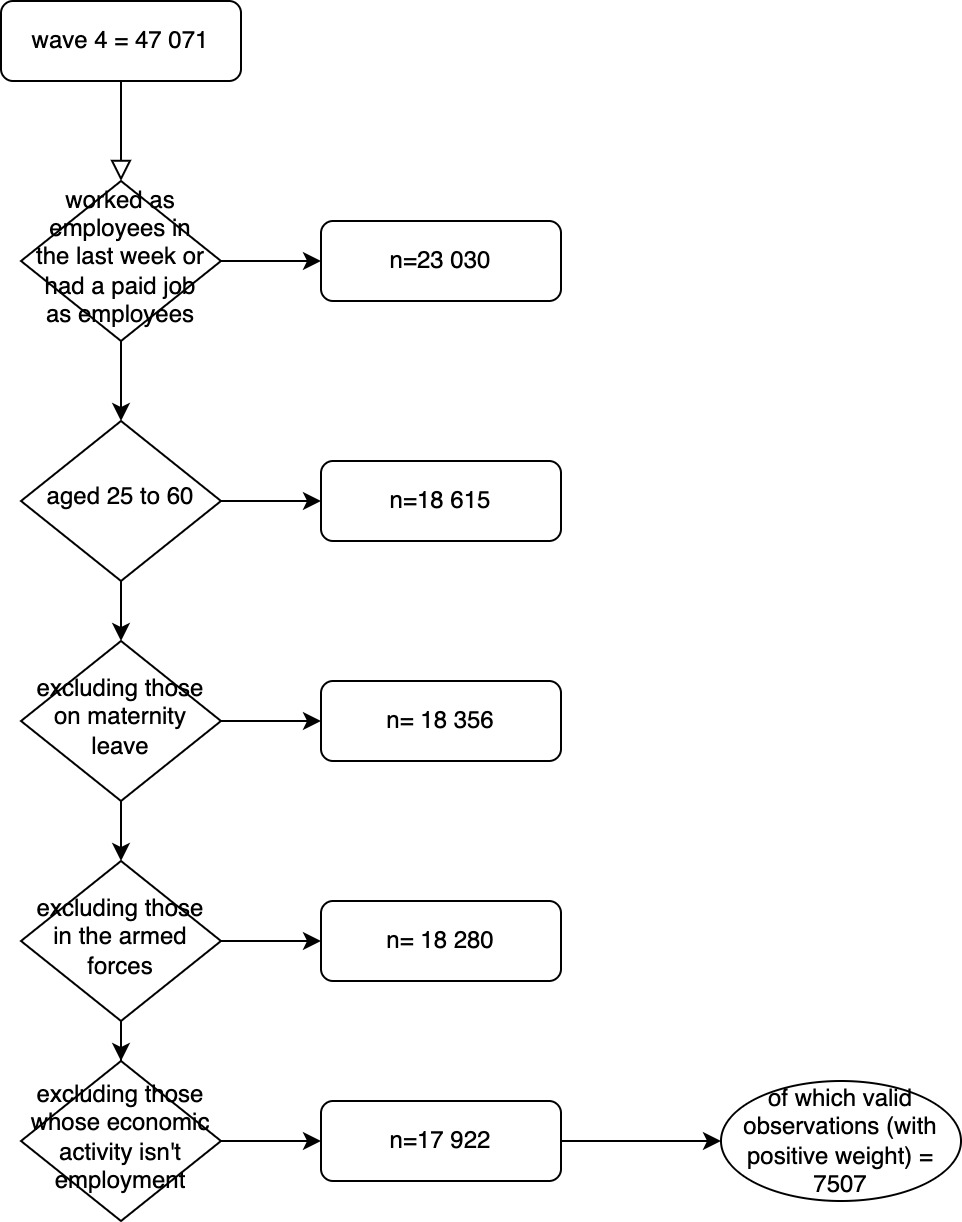


Figure S1 – Flow chart of sub-sample selection

# Descriptive table for sub-sample

Table S1 - Description of sub-sample for Latent Class Analysis. Note: weighted frequencies and wave 4 weight applied

| **Characteristic** | **N = 7,359**^1^ |
| --- | --- |
| Gender |  |
| Men | 3,657(50%) |
| Women | 3,702(50%) |
| Number of missing observations | 0 |
| ethnic background |  |
| White British | 6,216(85%) |
| White non-British | 410(5.6%) |
| Mixed | 107(1.5%) |
| Asian/Asian British | 430(5.9%) |
| Black/Black British | 161(2.2%) |
| Other | 29(0.4%) |
| Number of missing observations | 5 |
| Psychological distress (2/3) |  |
| No psychological distress | 5,915(80%) |
| Psychological distress | 1,439(20%) |
| Number of missing observations | 6 |
| Mental well-being (<19.3) |  |
| Moderate/high mental well-being | 5,680(77%) |
| Low mental well-being | 1,668(23%) |
| Number of missing observations | 10 |
| Age | 42(10) |
| ^1^n(%); Mean(SD) | |

# Latent Class Analysis - model selection


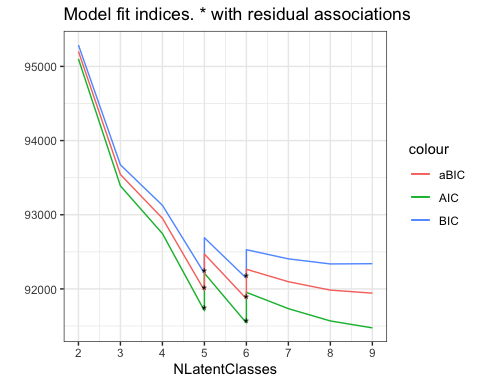


Figure S2 – Values of information criteria (on y axis) for k number of latent classes (x axis).

* denotes cluster solutions with residual associations

Table S2 - Overview and model fit indices for different k cluster solutions

| Number of latent classes | AIC | BIC | Vuong-Lo-Mendell-Rubin likelihood ratio test p value | Entropy | Residual associations? |
| --- | --- | --- | --- | --- | --- |
| 2 | 95,101.03 | 95,287.96 | 0.00 | 0.68 | No |
| 3 | 93,388.85 | 93,672.72 | 0.00 | 0.71 | No |
| 4 | 92,746.33 | 93,127.13 | 0.00 | 0.79 | No |
| 5 | 91,705.81 | 92,211.24 | 0.74 | 0.67 | Yes |
| 5 | 92,212.07 | 92,689.80 | 0.00 | 0.71 | No |
| 6 | 91,541.52 | 92,143.87 | 0.41 | 0.64 | Yes |
| 6 | 91,954.05 | 92,528.71 | 0.01 | 0.70 | No |
| 7 | 91,733.74 | 92,405.33 | 0.01 | 0.78 | No |
| 8 | 91,568.05 | 92,336.57 | 0.37 | 0.68 | No |
| 9 | 91,474.67 | 92,340.12 | 0.17 | 0.72 | No |

#

Table S3 - Item-response probabilities across manifest employment quality indicators. WT: Working time, PT=part-time, SER=Standard Employment Relationship

|  | Overall proportion | Precarious Unsustainable | High-Effort | Protected PT | Precarious Intensive | Portfolio | SER |
| --- | --- | --- | --- | --- | --- | --- | --- |
| **Proportions of clusters** |  | **0.18** | **0.11** | **0.15** | **0.18** | **0.16** | **0.22** |
| Permanent | 0.95 | 0.87 | 0.97 | 0.96 | 0.97 | 0.98 | 0.95 |
| Temporary | 0.05 | 0.13 | 0.03 | 0.04 | 0.03 | 0.01 | 0.05 |
| No Multiple jobholding | 0.93 | 0.92 | 0.89 | 0.95 | 0.93 | 0.96 | 0.92 |
| Multiple jobholding | 0.07 | 0.08 | 0.11 | 0.04 | 0.07 | 0.04 | 0.08 |
| Lowest pay quartile | 0.25 | 0.54 | 0.00 | 0.45 | 0.39 | 0.02 | 0.06 |
| 2nd/3rd pay quartile | 0.50 | 0.41 | 0.58 | 0.55 | 0.52 | 0.41 | 0.55 |
| Highest pay quartile | 0.25 | 0.05 | 0.42 | 0.00 | 0.10 | 0.57 | 0.39 |
| Non-wage benefit (pension) | 0.57 | 0.08 | 0.88 | 0.78 | 0.13 | 0.66 | 0.94 |
| No non-wage benefit (pension) | 0.43 | 0.92 | 0.12 | 0.22 | 0.87 | 0.34 | 0.06 |
| Pay progression | 0.36 | 0.12 | 0.57 | 0.43 | 0.15 | 0.26 | 0.63 |
| No pay progression | 0.64 | 0.88 | 0.43 | 0.57 | 0.85 | 0.74 | 0.37 |
| No unpaid overtime | 0.73 | 0.95 | 0.76 | 0.98 | 0.84 | 0.40 | 0.50 |
| Unpaid overtime | 0.27 | 0.05 | 0.24 | 0.02 | 0.16 | 0.60 | 0.50 |
| Not long hours | 0.84 | 1.00 | 0.62 | 0.99 | 0.67 | 0.81 | 0.88 |
| Long hours | 0.16 | 0.00 | 0.38 | 0.01 | 0.34 | 0.19 | 0.12 |
| No underemployment | 0.94 | 0.77 | 1.00 | 0.94 | 1.00 | 1.00 | 0.98 |
| Potential underemployment | 0.06 | 0.23 | 0.00 | 0.06 | 0.00 | 0.00 | 0.02 |
| Regular and standard WT | 0.71 | 0.74 | 0.15 | 0.74 | 0.48 | 0.95 | 0.95 |
| Irregular/non-standard WT | 0.29 | 0.26 | 0.85 | 0.26 | 0.52 | 0.05 | 0.05 |
| Informal WT flexibility | 0.64 | 0.50 | 0.48 | 0.51 | 0.64 | 0.88 | 0.73 |
| No informal WT flexibility | 0.36 | 0.50 | 0.52 | 0.49 | 0.36 | 0.12 | 0.27 |
| Recent training by employer | 0.29 | 0.12 | 0.45 | 0.25 | 0.25 | 0.26 | 0.42 |
| No recent training by employer | 0.71 | 0.88 | 0.55 | 0.75 | 0.75 | 0.74 | 0.58 |
| Collective representation | 0.50 | 0.19 | 0.87 | 0.83 | 0.10 | 0.02 | 0.99 |
| No collective representation | 0.50 | 0.81 | 0.13 | 0.17 | 0.91 | 0.98 | 0.01 |

Table S4 - Average Latent Class Probabilities for Most Likely Latent Class Membership (Row). SER=Standard Employment Relationship

|  | Precarious Unsustainable | High-Effort | Protected PT | Precarious Intensive | Portfolio | SER |
| --- | --- | --- | --- | --- | --- | --- |
|  |  |  |  |  |  |  |
| Precarious Unsustainable | 0.654 | 0.008 | 0.078 | 0.202 | 0.047 | 0.010 |
| High-Effort | 0.010 | 0.740 | 0.120 | 0.036 | 0.013 | 0.081 |
| Protected PT | 0.076 | 0.047 | 0.650 | 0.060 | 0.027 | 0.140 |
| Precarious Intensive | 0.182 | 0.031 | 0.037 | 0.696 | 0.052 | 0.002 |
| Portfolio | 0.046 | 0.015 | 0.031 | 0.058 | 0.842 | 0.008 |
| SER | 0.007 | 0.049 | 0.114 | 0.006 | 0.013 | 0.811 |

# Characteristics of clusters

Table S5 - Occupational and socio-demographic characteristics of EQ clusters. Note: weighted frequencies and wave 5 weight applied, sub-sample with complete information

| **Characteristic** | **SER**, N = 1,498^1^ | **Precarious Unsustainable**, N = 1,180^1^ | **High-Effort**, N = 673^1^ | **Protected PT**, N = 821^1^ | **Precarious Intensive**, N = 966^1^ | **Portfolio**, N = 989^1^ |
| --- | --- | --- | --- | --- | --- | --- |
| **Gender** |  |  |  |  |  |  |
| Men | 706(47%) | 474(40%) | 425(63%) | 224(27%) | 534(55%) | 632(64%) |
| Women | 792(53%) | 706(60%) | 247(37%) | 597(73%) | 432(45%) | 357(36%) |
| **Educational attainment** |  |  |  |  |  |  |
| Degree | 1,025(68%) | 361(31%) | 341(51%) | 253(31%) | 284(29%) | 641(65%) |
| A-level etc. | 254(17%) | 236(20%) | 154(23%) | 204(25%) | 221(23%) | 170(17%) |
| GSCE etc. | 160(11%) | 338(29%) | 128(19%) | 227(28%) | 287(30%) | 131(13%) |
| Other/no qualification | 58(3.8%) | 245(21%) | 50(7.4%) | 137(17%) | 173(18%) | 46(4.7%) |
| **Ethnic background** |  |  |  |  |  |  |
| White British | 1,291(86%) | 980(83%) | 566(84%) | 728(89%) | 832(86%) | 836(85%) |
| White Non-British | 75(5.0%) | 83(7.1%) | 34(5.0%) | 17(2.0%) | 54(5.6%) | 65(6.6%) |
| Mixed/other | 44(3.0%) | 17(1.5%) | 11(1.7%) | 9(1.1%) | 16(1.6%) | 17(1.8%) |
| Asian/Asian British | 56(3.7%) | 68(5.8%) | 49(7.3%) | 53(6.5%) | 51(5.2%) | 58(5.9%) |
| Black/Black British | 31(2.1%) | 31(2.6%) | 13(1.9%) | 14(1.7%) | 14(1.4%) | 12(1.2%) |
| **Occupational group (SOC2000)** |  |  |  |  |  |  |
| Managers and Senior Officials | 263(18%) | 96(8.2%) | 63(9.4%) | 44(5.4%) | 173(18%) | 410(42%) |
| Professional Occupations | 462(31%) | 71(6.1%) | 103(15%) | 48(5.9%) | 29(3.0%) | 190(19%) |
| Associate Professional and Technical Occupations | 324(22%) | 117(10%) | 205(30%) | 101(12%) | 67(7.0%) | 154(16%) |
| Administrative and Secretarial Occupations | 217(15%) | 202(17%) | 31(4.6%) | 152(19%) | 72(7.6%) | 105(11%) |
| Skilled Trades Occupations | 63(4.2%) | 114(9.7%) | 41(6.1%) | 38(4.6%) | 95(9.9%) | 40(4.1%) |
| Personal Service Occupations | 96(6.4%) | 135(12%) | 55(8.2%) | 163(20%) | 154(16%) | 19(1.9%) |
| Sales and Customer Service Occupations | 20(1.4%) | 125(11%) | 19(2.9%) | 94(11%) | 122(13%) | 16(1.7%) |
| Process, Plant and Machine Operatives | 32(2.1%) | 98(8.4%) | 81(12%) | 53(6.5%) | 101(11%) | 29(2.9%) |
| Elementary Occupations | 20(1.3%) | 212(18%) | 74(11%) | 125(15%) | 142(15%) | 20(2.0%) |
| Number of missing observations | 1 | 10 | 0 | 2 | 11 | 6 |
| **Length of prior unemployment (months)** | 4 (21) | 7 (24) | 4 (20) | 6 (27) | 8 (26) | 3 (16) |
| **Age** | 43 (10) | 42 (10) | 42 (9) | 44 (10) | 41 (10) | 42 (9) |
| **Main working hours** | 35 (6) | 28 (12) | 39 (8) | 28 (9) | 36 (12) | 38 (6) |
| Number of missing observations | 2 | 5 | 1 | 2 | 4 | 0 |
| **Total working hours** | 40 (8) | 29 (12) | 46 (12) | 30 (10) | 42 (15) | 43 (9) |
| Number of missing observations | 2 | 5 | 1 | 2 | 4 | 0 |
| autonomy over job tasks |  |  |  |  |  |  |
| A lot/some | 1,205(80%) | 740(63%) | 445(66%) | 507(62%) | 708(73%) | 872(88%) |
| A little/None | 292(20%) | 438(37%) | 227(34%) | 314(38%) | 258(27%) | 116(12%) |
| Number of missing observations | 0 | 1 | 0 | 0 | 0 | 0 |
| autonomy over work pace |  |  |  |  |  |  |
| A lot/some | 1,176(79%) | 811(69%) | 457(68%) | 551(67%) | 733(76%) | 869(88%) |
| A little/None | 321(21%) | 369(31%) | 215(32%) | 269(33%) | 233(24%) | 119(12%) |
| Number of missing observations | 0 | 0 | 0 | 1 | 0 | 0 |
| autonomy over work manner |  |  |  |  |  |  |
| A lot/some | 1,336(89%) | 907(77%) | 520(77%) | 619(75%) | 811(84%) | 940(95%) |
| A little/None | 161(11%) | 273(23%) | 153(23%) | 202(25%) | 155(16%) | 48(4.8%) |
| Number of missing observations | 0 | 0 | 0 | 1 | 0 | 1 |
| autonomy over task order |  |  |  |  |  |  |
| A lot/some | 1,340(89%) | 875(74%) | 495(74%) | 591(72%) | 776(81%) | 946(96%) |
| A little/None | 158(11%) | 305(26%) | 177(26%) | 229(28%) | 188(19%) | 42(4.3%) |
| Number of missing observations | 0 | 0 | 0 | 1 | 1 | 0 |
| autonomy over work hours |  |  |  |  |  |  |
| A lot/some | 913(61%) | 368(31%) | 158(23%) | 214(26%) | 390(40%) | 666(67%) |
| A little/None | 584(39%) | 810(69%) | 515(77%) | 607(74%) | 575(60%) | 323(33%) |
| Number of missing observations | 0 | 1 | 0 | 0 | 0 | 0 |
| ^1^n(%); Mean (SD) | | | | | | |

# Goodness of fit for regression models

Table S6 - List of p-values of Hosmer-Lemeshow goodness of fit tests for regression models, GHQ=psychological distress, MWB=low mental well-being

|  | Men | Women |  |  |
| --- | --- | --- | --- | --- |
| Age adjusted EQ GHQ | 0.017 | 0.21 |  |  |
| Age adjusted past UE GHQ | 0.77 | 0.33 |  |  |
| Dose-response UE GHQ | 0.79 | 0.66 |  |  |
| Adjusted GHQ | 0.38 | 0.19 |  |  |
| Full model GHQ | 0.65 | 0.23 |  |  |
| Age adjusted EQ | 0.50 | 0.77 |  |  |
| Age adjusted past UE MWB | 0.087 | 0.75 |  |  |
| Dose-response UE MWB | 0.19 | 0.32 |  |  |
| Adjusted MWB | 0.35 | 0.016 |  |  |
| Full model MWB | 0.66 | 0.025 |  |  |

# Sensitivity analyses

Table S7 - Regression results. Sensitivity analyses with GHQ score 3/4 - Men

|  | **Psychological distress** | | | | |
| --- | --- | --- | --- | --- | --- |
|  | *Model 1* | *Model 2* | *Model 3* | *Model 4* | *Model 5* |
| **EQ type (REF: SER)** | **-** | **-** | **-** | **-** | **-** |
| Precarious Unsustainable | 1.23 (0.81, 1.86) |  |  | 1.30 (0.83, 2.02) | 1.29 (0.82, 2.01) |
| High-Effort | 1.24 (0.82, 1.88) |  |  | 1.30 (0.86, 1.98) | 1.31 (0.86, 2.00) |
| Protected Part-time | 0.90 (0.54, 1.48) |  |  | 0.96 (0.57, 1.62) | 0.95 (0.56, 1.60) |
| Precarious Intensive | 0.83 (0.55, 1.27) |  |  | 0.89 (0.56, 1.40) | 0.87 (0.55, 1.38) |
| Portfolio | 0.59* (0.39, 0.90) |  |  | 0.56** (0.37, 0.86) | 0.56** (0.37, 0.86) |
| **Been unemployed in the past (Ref: No)** | **-** | **-** | **-** | **-** | **-** |
| Yes |  | 1.22 (0.91, 1.63) | 1.17 (0.84, 1.62) |  | 1.21 (0.90, 1.62) |
| **Past unemployment (in months)** |  |  | 1.00 (1.00, 1.01) |  |  |
| Observations | 2565 | 2565 | 2565 | 2565 | 2565 |
| Odds ratios (and 95% confidence intervals). | | | | | |
| All models are adjusted by age, and Models 4 and 5 are additionally adjusted by partnership status, ethnic background, and educational attainment | | | | | |
| *p<0.05, **p<0.01, ***p<0.001 | | | | | |

Table S8 - Regression results. Sensitivity analyses with GHQ score 3/4 - Women

|  | **Psychological distress** | | | | |
| --- | --- | --- | --- | --- | --- |
|  | *Model 1* | *Model 2* | *Model 3* | *Model 4* | *Model 5* |
| **EQ type (REF: SER)** | **-** | **-** | **-** | **-** | **-** |
| Precarious Unsustainable | 0.85 (0.64, 1.14) |  |  | 0.93 (0.68, 1.26) | 0.92 (0.68, 1.25) |
| High-Effort | 1.13 (0.80, 1.60) |  |  | 1.17 (0.82, 1.66) | 1.18 (0.83, 1.67) |
| Protected Part-time | 1.03 (0.79, 1.35) |  |  | 1.11 (0.83, 1.48) | 1.11 (0.83, 1.47) |
| Precarious Intensive | 1.20 (0.87, 1.65) |  |  | 1.28 (0.91, 1.78) | 1.27 (0.90, 1.78) |
| Portfolio | 1.05 (0.76, 1.46) |  |  | 1.07 (0.77, 1.49) | 1.06 (0.76, 1.49) |
| **Been unemployed in the past (Ref: No)** | **-** | **-** | **-** | **-** | **-** |
| Yes |  | 1.22 (0.96, 1.56) | 1.33* (1.02, 1.74) |  | 1.19 (0.92, 1.52) |
| **Past unemployment (in months)** |  |  | 1.00 (0.99, 1.00) |  |  |
| Observations | 3704 | 3704 | 3704 | 3704 | 3704 |
| Odds ratios (and 95% confidence intervals). | | | | | |
| All models are adjusted by age, and Models 4 and 5 are additionally adjusted by partnership status, ethnic background, and educational attainment | | | | | |
| *p<0.05, **p<0.01, ***p<0.001 | | | | | |

Table S9 - Sensitivity analyses. Lower SWEMBS cut-off. Men

|  | **Low mental well-being** | | | | |
| --- | --- | --- | --- | --- | --- |
|  | *Model 1* | *Model 2* | *Model 3* | *Model 4* | *Model 5* |
| **EQ type (REF: SER)** | **-** | **-** | **-** | **-** | **-** |
| Precarious Unsustainable | 2.21*** (1.46, 3.36) |  |  | 1.95** (1.25, 3.06) | 1.94** (1.23, 3.04) |
| High-Effort | 1.49 (0.96, 2.32) |  |  | 1.41 (0.91, 2.18) | 1.41 (0.91, 2.19) |
| Protected Part-time | 2.22** (1.32, 3.72) |  |  | 1.94* (1.14, 3.29) | 1.93* (1.14, 3.27) |
| Precarious Intensive | 1.57* (1.05, 2.36) |  |  | 1.40 (0.90, 2.16) | 1.38 (0.89, 2.13) |
| Portfolio | 0.62* (0.39, 0.99) |  |  | 0.60* (0.37, 0.96) | 0.6* (0.37, 0.96) |
| **Been unemployed in the past (Ref: No)** | **-** | **-** | **-** | **-** | **-** |
| Yes |  | 1.29 (0.97, 1.71) | 1.33 (0.98, 1.81) |  | 1.15 (0.86, 1.54) |
| **Past unemployment (in months)** |  |  | 1.00 (0.99, 1.01) |  |  |
| Observations | 2565 | 2565 | 2565 | 2565 | 2565 |
| Odds ratios (and 95% confidence intervals). | | | | | |
| All models are adjusted by age, and Models 4 and 5 are additionally adjusted by partnership status, ethnic background, and educational attainment | | | | | |
| *p<0.05, **p<0.01, ***p<0.001 | | | | | |

Table S10 - Sensitivity analyses. Lower SWEMBS cutoff. Women

|  | **Low mental well-being** | | | | |
| --- | --- | --- | --- | --- | --- |
|  | *Model 1* | *Model 2* | *Model 3* | *Model 4* | *Model 5* |
| **EQ type (REF: SER)** | **-** | **-** | **-** | **-** | **-** |
| Precarious Unsustainable | 1.82*** (1.33, 2.5) |  |  | 1.62** (1.16, 2.27) | 1.61** (1.15, 2.26) |
| High-Effort | 1.13 (0.73, 1.75) |  |  | 1.11 (0.71, 1.73) | 1.12 (0.72, 1.75) |
| Protected Part-time | 1.71*** (1.24, 2.34) |  |  | 1.52* (1.08, 2.13) | 1.51* (1.08, 2.13) |
| Precarious Intensive | 2.02*** (1.45, 2.83) |  |  | 1.75** (1.25, 2.45) | 1.74** (1.24, 2.44) |
| Portfolio | 0.95 (0.60, 1.49) |  |  | 0.91 (0.58, 1.44) | 0.91 (0.57, 1.44) |
| **Been unemployed in the past (Ref: No)** | **-** | **-** | **-** | **-** | **-** |
| Yes |  | 1.27 (0.98, 1.65) | 1.27 (0.96, 1.69) |  | 1.21 (0.92, 1.59) |
| **Past unemployment (in months)** |  |  | 1.00 (1.00, 1.00) |  |  |
| Observations | 3704 | 3704 | 3704 | 3704 | 3704 |
| Odds ratios (and 95% confidence intervals). | | | | | |
| All models are adjusted by age, and Models 4 and 5 are additionally adjusted by partnership status, ethnic background, and educational attainment | | | | | |
| *p<0.05, **p<0.01, ***p<0.001 | | | | | |

|  | **Psychological distress** | | | | **Low-mental wellbeing** | | | |
| --- | --- | --- | --- | --- | --- | --- | --- | --- |
|  | *Model 1* | *Model 6* | *Model 1* | *Model 6* | *Model 1* | *Model 6* | *Model 1* | *Model 6* |
| **Employment quality type (REF: Standard Employment Relationship)** | **-** | **-** | **-** | **-** | **-** | **-** | **-** | **-** |
| Precarious Unsustainable |  | 1.27 (0.86, 1.87) |  | 1.27 (0.86, 1.87) |  | 1.53* (1.05, 2.23) |  | 1.54* (1.06, 2.24) |
| High-Effort |  | 1.18 (0.81, 1.72) |  | 1.18 (0.81, 1.72) |  | 1.33 (0.94, 1.9) |  | 1.33 (0.94, 1.89) |
| Protected Part-time |  | 1.07 (0.66, 1.74) |  | 1.07 (0.66, 1.73) |  | 1.69* (1.07, 2.65) |  | 1.69* (1.08, 2.66) |
| Precarious Intensive |  | 0.94 (0.64, 1.39) |  | 0.94 (0.64, 1.39) |  | 1.11 (0.76, 1.61) |  | 1.11 (0.76, 1.62) |
| Portfolio |  | 0.65* (0.45, 0.93) |  | 0.65* (0.45, 0.93) |  | 0.68* (0.47, 0.96) |  | 0.68* (0.48, 0.96) |
| **Over 6 months unemployment (ref: 6 months or less of unemployment)** | **-** | **-** | **-** | **-** | **-** | **-** | **-** | **-** |
| Over 6 months past of unemployment | 0.9 (0.66, 1.24) | 0.88 (0.64, 1.20) |  |  | 1.41* (1.06, 1.86) | 1.25 (0.93, 1.67) |  |  |
| **Over 12 months unemployment (ref: 12 months or less of unemployment)** | **-** | **-** | **-** | **-** | **-** | **-** | **-** | **-** |
| Over 12 months of past unemployment |  |  | 0.88 (0.59, 1.31) | 0.85 (0.57, 1.27) |  |  | 1.52* (1.08, 2.15) | 1.32 (0.92, 1.88) |
| Observations | 2565 | 2565 | 2565 | 2565 | 2565 | 2565 | 2565 | 2565 |
| Odds ratios (and 95% confidence intervals). | | | | | | | | |
| Model 1 is adjusted by age, and Model 6 is additionally adjusted by partnership status, ethnic background, and educational attainment | | | | | | | | |
| *p<0.05, **p<0.01, ***p<0.001 | | | | | | | | |

Table S11 Associations between employment quality types, past unemployment, and psychological distress and mental wellbeing among men. Odds ratios (and 95% confidence intervals) from logistic regressions

Table S12 - Associations between employment quality types, past unemployment, and psychological distress and mental wellbeing among women. Odds ratios (and 95% confidence intervals) from logistic regressions

|  | **Psychological distress** | | | | **Low-mental wellbeing** | | | |
| --- | --- | --- | --- | --- | --- | --- | --- | --- |
|  | *Model 1* | *Model 6* | *Model 1* | *Model 6* | *Model 1* | *Model 6* | *Model 1* | *Model 6* |
| **Employment quality type (REF: Standard Employment Relationship)** | **-** | **-** | **-** | **-** | **-** | **-** | **-** | **-** |
| Precarious Unsustainable |  | 0.85 (0.64, 1.12) |  | 0.85 (0.64, 1.12) |  | 1.38* (1.04, 1.81) |  | 1.37* (1.04, 1.81) |
| High-Effort |  | 1.06 (0.77, 1.47) |  | 1.06 (0.76, 1.46) |  | 1.14 (0.81, 1.62) |  | 1.13 (0.8, 1.6) |
| Protected Part-time |  | 0.99 (0.76, 1.29) |  | 0.99 (0.76, 1.29) |  | 1.44** (1.09, 1.9) |  | 1.43* (1.09, 1.89) |
| Precarious Intensive |  | 1.13 (0.83, 1.54) |  | 1.13 (0.83, 1.54) |  | 1.43* (1.07, 1.92) |  | 1.43* (1.07, 1.91) |
| Portfolio |  | 1.00 (0.74, 1.35) |  | 1.00 (0.74, 1.35) |  | 1.01 (0.72, 1.42) |  | 1.02 (0.73, 1.43) |
| **Over 6 months unemployment (ref: 6 months or less of unemployment)** | **-** | **-** | **-** | **-** | **-** | **-** | **-** | **-** |
| Over 6 months past of unemployment | 1.26 (0.96, 1.65) | 1.22 (0.93, 1.62) |  |  | 1.55** (1.18, 2.03) | 1.45** (1.10, 1.91) |  |  |
| **Over 12 months unemployment (ref: 12 months or less of unemployment)** | **-** | **-** | **-** | **-** | **-** | **-** | **-** | **-** |
| Over 12 months of past unemployment |  |  | 1.18 (0.85, 1.65) | 1.16 (0.83, 1.63) |  |  | 1.73*** (1.25, 2.4) | 1.56* (1.11, 2.19) |
| Observations | 3704 | 3704 | 3704 | 3704 | 3704 | 3704 | 3704 | 3704 |
| Odds ratios (and 95% confidence intervals). | | | | | | | | |
| Model 1 is adjusted by age, and Model 6 is additionally adjusted by partnership status, ethnic background, and educational attainment | | | | | | | | |
| *p<0.05, **p<0.01, ***p<0.001 | | | | | | | | |

Table S13 - Regression results - Men - percentage UE

|  | **Psychological distress** | | **Low-mental wellbeing** | |
| --- | --- | --- | --- | --- |
|  | % unemployment | Model 5 | % unemployment | Model 5 |
| Precarious Unsustainable |  | 1.26 (0.85, 1.87) |  | 1.6* (1.10, 2.33) |
| High-Effort |  | 1.20 (0.82, 1.76) |  | 1.33 (0.93, 1.89) |
| Protected Part-time |  | 1.09 (0.67, 1.77) |  | 1.72* (1.09, 2.72) |
| Precarious Intensive |  | 0.94 (0.64, 1.39) |  | 1.11 (0.76, 1.63) |
| Portfolio |  | 0.66* (0.46, 0.95) |  | 0.67* (0.47, 0.95) |
| Unemployment (% of career) | 1.00 (0.99, 1.02) | 1.00 (0.99, 1.02) | 1.00 (0.99, 1.02) | 1.00 (0.99, 1.01) |
| Observations | 2536 | 2536 | 2536 | 2536 |
| Odds ratios (and 95% confidence intervals). | | | | |
| Model ‘% unemployment’ is adjusted by age, and Model 5 is additionally adjusted by partnership status, ethnic background, and educational attainment | | | | |
| *p<0.05, **p<0.01, ***p<0.001 | | | | |

Table S14 - Regression results - Women - percentage UE

|  | **Psychological distress** | | **Low-mental wellbeing** | |
| --- | --- | --- | --- | --- |
|  | % unemployment | Model 5 | % unemployment | Model 5 |
| Precarious Unsustainable |  | 0.83 (0.62, 1.10) |  | 1.41* (1.06, 1.87) |
| High-Effort |  | 1.02 (0.73, 1.42) |  | 1.16 (0.82, 1.65) |
| Protected Part-time |  | 0.98 (0.75, 1.29) |  | 1.49** (1.12, 1.97) |
| Precarious Intensive |  | 1.11 (0.81, 1.52) |  | 1.50** (1.12, 2.01) |
| Portfolio |  | 0.99 (0.73, 1.34) |  | 1.06 (0.75, 1.48) |
| Unemployment (% of career) | 1.00 (0.99, 1.01) | 1.00 (0.99, 1.01) | 1.01* (1.00, 1.02) | 1.01 (1.00, 1.02) |
| Observations | 3647 | 3647 | 3647 | 3647 |
| Odds ratios (and 95% confidence intervals). | | | | |
| Model ‘% unemployment’ is adjusted by age, and Model 5 is additionally adjusted by partnership status, ethnic background, and educational attainment | | | | |
| *p<0.05, **p<0.01, ***p<0.001 | | | | |
